# Clinician and patient experiences with shared decision-making to promote daily arm use for individuals with chronic stroke: an exploratory qualitative study

**DOI:** 10.3389/fresc.2024.1414878

**Published:** 2024-09-19

**Authors:** Amanda Gahlot, Grace Richardson, Patricia Librea, Grace J. Kim

**Affiliations:** ^1^ Department of Occupational Therapy, NYU Steinhardt School of Culture, Education, and Human Development, New York, NY, United States; ^2^ Department of Rehabilitation Medicine, NYU Langone Health, New York, NY, United States

**Keywords:** motivational interviewing, stroke rehabilitation, shared decision making, telehealth, upper extremity

## Abstract

**Purpose:**

To explore the attitudes and experiences of clinicians and individuals with chronic stroke on the use of shared decision-making (SDM) during upper extremity rehabilitation to improve daily arm use in the home environment. Specifically, we aimed to describe clinician and client perspectives regarding the facilitators and barriers to using SDM within the context of a self-directed upper extremity intervention for individuals living in the community with chronic stroke.

**Methods:**

Data were collected within the context of an interventional study examining the feasibility of the Use My Arm-Remote intervention. Focus group interviews were conducted with the clinicians (*n* = 3) providing the intervention and individual semi-structured interviews with the participants (*n* = 15) of the study. All interview data were collected after the end of the intervention period. Data were analyzed using thematic analysis.

**Results:**

The following themes were identified: (1) Equal partnership; (2) Enhancing clinician confidence; and (3) This is different. Facilitators and barriers were identified within each theme. Key facilitators for clinicians were competence with SDM and patient characteristics; while facilitators for patients were open and trusting relationships with clinicians and personalized experience. Key barriers to SDM for clinicians were lack of expertise in SDM and participant buy in; while patients identified a lack of foundational knowledge of stroke rehabilitation as a potential barrier.

**Conclusions:**

Key barriers were analyzed using the consolidated framework for advancing implementation science to interpret results and identify strategies for enhancing the implementation of SDM in a virtual setting. The CFIR-ERIC tool highlighted the need for targeted educational meetings and materials to address the training and educational needs of both clinicians and patients for future iterations of this intervention.

## 1 Introduction

Shared decision making (SDM) is defined as a collaborative communication process between patients, families, and clinicians where patient values and preferences are prioritized along with existing evidence when making decisions about a specific scenario (1). In a recent scientific statement, the American Heart Association endorsed the use of SDM in healthcare delivery and cardiovascular guidelines stating that SDM is “critical” to both promoting healthy outcomes and enhancing equity within healthcare (2). Strategies to implement SDM in cardiovascular care include clinician training, inclusion of multi-disciplinary team, and use of patient decision aid tools (3–5). SDM was supported by the US Affordable Care Act in 2010, has been studied in the context of chronic health conditions such as cancer, kidney disease, and cardiovascular illness, and has demonstrated improved patient involvement, knowledge, health outcomes, and patient-centered care (2, 6, 7). However, applying SDM to stroke rehabilitation is an emerging area of study (1, 8, 9). As stroke can cause chronic impairment, requiring intensive and extended rehabilitation, SDM may be particularly beneficial as it may also enhance adherence and motivation (10).

The limited but growing evidence on the use of SDM in stroke rehabilitation have used patient decision tools and motivational interviewing as strategies to implement SDM. Ikegami et al. (11) used a patient decision aid tool in the acute stroke population to implement SDM and concluded that the SDM group demonstrated both improved upper extremity (UE) motor function and improved performance with activities of daily living compared to the control group. Another strategy to implement SDM is motivational interviewing (MI), which provides clinicians with the needed *communication skills* to support the process of SDM. MI is a counseling approach based on the guiding principle that behavior change is contingent on the client, rather than the clinician, to drive decisions and identify reasons for change (12). Watkins et al. (13) found that MI counseling with individuals in the acute stage of stroke was associated with improved mood compared to usual care. Furthermore, a meta-analysis of MI interventions after stroke concluded that MI may improve depression and quality of life of stroke patients (14).

Previous studies using MI to implement SDM have typically focused on in-person interventions to address mood, acceptance and adjustment, and lifestyle changes (14–16). However, the Use My Arm-Remote (UMA-R) (17, 18) intervention used MI to implement SDM in a virtual setting to directly promote self-directed arm training in functional tasks in the home environment. The use of SDM to promote self-directed arm training in the home setting is a novel application in stroke rehabilitation. The use of SDM may be a key component for home-based self-directed arm training interventions in individuals with stroke. Decreased patient motivation and adherence are identified limitations in both traditional home exercise programs (19–21) and emerging self-directed interventions because they require patients to complete therapy activities independently under indirect supervision of a clinician (22). The use of a SDM approach may optimize training adherence because it facilitates client engagement and decision-making about treatment goals and activities (1). SDM can be implemented in UE stroke rehabilitation through a collaborative process between the clinician and the patient to promote patients to identify self-valued functional goals, identify and address barriers to UE training on their own, and add or modify training goals with support from the clinician throughout the intervention period.

While initial studies using SDM in UE stroke rehabilitation interventions appear promising, there is lack of research on the underlying factors that either support or hinder its implementation (23). Understanding the practical implications of a successful intervention is critical to ensure it can be applied outside of a research setting (24). For example, although constraint-induced movement therapy (CIMT) is the most widely examined intervention to demonstrate a strong level of evidence for UE recovery (25), CIMT has not been successfully implemented in non-research settings because facilitators and barriers were not considered during the design stage (26, 27). Therefore, there is a need to develop foundational knowledge that incorporates the perspectives of both clinicians and patients with experience engaging in SDM to better understand how to effectively implement SDM into UE rehabilitation interventions and ultimately into clinical practice (2). As clinicians and patients have different perspectives on facilitators and barriers to successful implementation of SDM (28, 29), this qualitative study aimed to explore the attitudes and experiences of both groups to identify the facilitators and barriers to integrating SDM within the context of UE rehabilitation for individuals living the community with chronic stroke.

## 2 Methods

A descriptive qualitative design using an exploratory approach was used to capture the perspectives of therapists and individuals with stroke who participated in a self-directed UE intervention to better understand SDM within the context of UE stroke rehabilitation. A qualitative descriptive design recognizes the subjective nature of individuals’ experiences and allows researchers to explore the phenomenon from the perspective of those experiencing it (30). This qualitative study took place as part of a larger feasibility study of the UMA-R intervention (18).

### 2.1 Participants and recruitment

Occupational therapists (OTs) (*n* = 3) with experience in stroke rehabilitation were selected for training and implementation of SDM. Each of the three OTs had advanced training in working with the neurologic population and over 15 years of clinical experience. Additionally, all were in a supervisory position. Semi-structured interview data were collected from all study participants completing the primary study (*n* = 15). Kim et al. (18) reports specific recruitment, inclusion and exclusion criteria, and demographics of stroke participants. Overall, stroke participants were recruited from outpatient clinics, support groups, and a stroke research database, were living in the community at the time of intervention, and were at least 6 months post injury with mean time since injury 4.5 years.

### 2.2 Intervention

SDM was implemented during the Use My Arm Remote (UMA-R) program to create personalized UE training plan for participants (17). In Phase 1, study clinicians received MI training in order develop communication skills that support SDM. This training included two 3-h training sessions completed prior to the start of the study and one booster session that was completed once clinicians had experience engaging in MI with at least one patient in the study. MI training consisted of both didactic materials and hands-on activities using shared decision-making strategies geared toward having the clinicians facilitate patients to drive and create their own UE training goals and treatment plan. Phase 2 consisted of 3–6 meetings between the clinicians and the patients via Zoom. These meetings provided opportunities for the patients to learn about the UMA-R program, to identify potential barriers to UE self-training and to tailor their treatment plans and develop personalized goals using SDM principles. In this phase, patients worked with clinicians to enhance their confidence and readiness to engage in self-training. In Phase 3, patients implemented their personalized UE training plan over a 4-week training period. Patients also completed weekly check-ins with the clinicians over Zoom, whereby they continued to collaborate with the therapist about their performance and problem-solved challenges they encountered, and update the treatment plan as needed (17).

### 2.3 Data collection

The patients completed individual semi-structured interviews within 1 week of completing the 4-week intervention. Each interview lasted approximately 1 h and were completed by the principal investigator of the study (GK). The clinicians completed two 1-h focus group sessions led by GK. Author AG was present for clinician focus group interviews to observe and take field notes, which were used in creating codes. Semi-structured interviews and focus groups were completed via Zoom by author GK who was not involved in delivering the intervention. An interview guide was developed consisting of a series of questions and probes, designed to elicit the perspectives of clinicians and patients regarding the remote delivery of intervention, facilitators and barriers to delivery of SDM in a virtual setting, and perceptions of SDM compared to traditional therapy interventions. See Table 1 for a semi-structured interview guide and focus group guide. Ethical approval was granted by IRB through NYU Langone Health (IRB #i21-00513) prior to the commencement of data collection.

**Table 1:** Patient and clinician semi-structured interview guides.

Patient interview
General impressions: Tell me about your experience participating in the use my arm protocol?
Education What did you think about the educational materials? Was this helpful in any way to help you participate in in the arm training? If so, how? What does recovery mean to you? How much effort do you think it takes to create change in your arm ability?
Motivational interviewing How do you feel about being able to select your own goal and tasks in your practice? Can you describe the experience of selecting your own goals and activities? How did this process affect your participation in the arm training? Do you think we should keep doing this?
Weekly check-ins How was the weekly check-in with the therapist? What was most helpful? Least helpful? Is this something you recommend we keep for next time?
Refinement Is there anything you would change about creating your own goals and tasks to practice? How do you feel about continued rehabilitation on your own?
Conclusion Is there anything else you would like to tell me about your experience with the Use My Arm program?
Clinician interview
MI training How did the MI training prepare you for implementing a SDM approach with your clients? How did the booster session contribute? What aspects of the MI training was most helpful? Least helpful? How did the weekly huddle inform your SDM approach? What would you have liked more of during the study that may have helped you implement MI informed SDM? What questions do you have now about the SDM approach? How can we improve the training procedures next time?
Implementation How comfortable do you feel using SDM moving forward? How does SDM approach fit into your clinical practice? What resources do you need to integrate this into standard practice?
General Who is the ideal candidate for this type of study? What other aspects of the participant should be considered for exclusion in the study?

### 2.4 Data analysis

Interviews were transcribed verbatim and the research team familiarized themselves with the content of the transcripts to gain an overall understanding of the data (31, 32). Individual and group interview data were coded using MAXQDA software (33). Three of the authors completed two rounds of coding by highlighting segments of the transcripts that were deemed meaningful or relevant to the research question using inductive coding techniques (31, 32). These inductive codes were the starting point for the development of meaning, and were added to the code book. Deductive coding, informed by our study aim, was completed by viewing these set codes as the conceptual ideas through which the authors sought to understand through the dataset (34). Deductive coding and summaries were completed separately to enhance rigor. Codes were analyzed using reflexive thematic analysis (31). The authors collaboratively explored areas where there were similarities of meaning between the codes and grouped them into themes. Thematic maps were used to consider how provisional themes captured the centrality of the research question, as well as how they were related to one another. The emerging themes developed into parent themes and sub-themes, highlighting arising concepts and ideas, and these themes were cross-referenced between the authors (31).

### 2.5 Author positionality

The following beliefs, assumptions, and experiences associated with the research team contributed to the understanding and interpretation of the data (35). All authors are OTs who have clinical and research experience in stroke rehabilitation. The authors believe that each lived experience is unique to the participant of the study and stroke survivors are their own experts in their recovery. All authors prioritize client-centered care and partnership between clinicians and clients. Lastly, while all authors have experience working with stroke survivors, they do not have personal working knowledge of recovery from stroke.

### 2.6 Rigor

The research team followed guidelines to establish trustworthiness and rigor in the data collection and analysis process (36, 37). To enhance rigor and transparency, written records were kept during all stages of data collection and analysis including retention of a record of codes and interpretations. Reflexivity was addressed through author positionality and author reflections during analysis. Credibility was marked by independent coding by three authors (AG, GR, and PL) and triangulation through discussion including the fourth author (GK). The use of MAXQDA software enhanced accuracy and organization of the coding and analysis processes, allowing for the integration of each author's comments and individual codes in the development and management of coded summaries. Lastly, participant characteristics and sample interview questions are provided to allow readers to judge transferability to other settings.

## 3 Results

**Figure 1:**
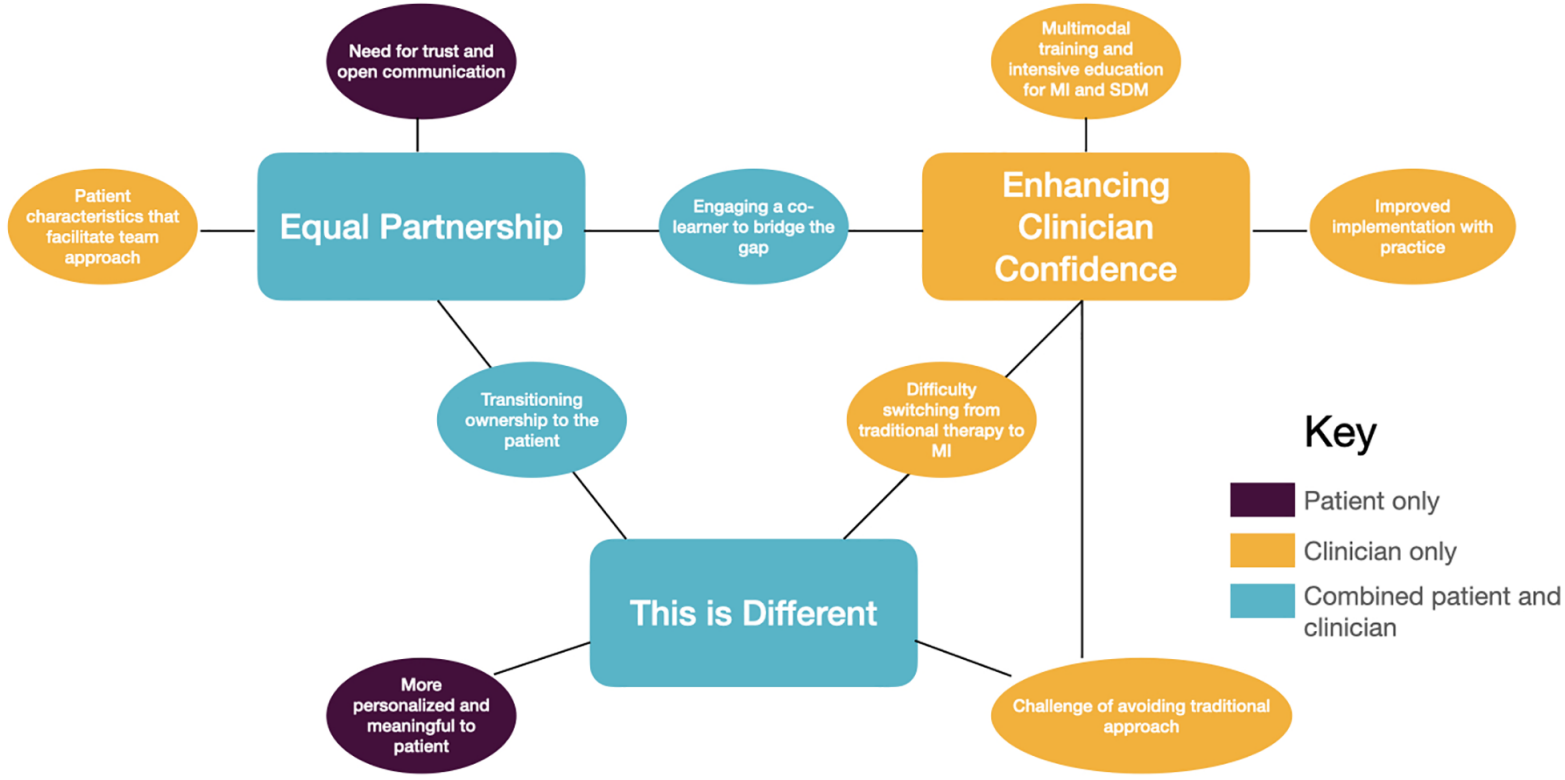
Thematic map for implementation of shared decision making with chronic stroke survivors for upper extremity recovery.

Three main themes were identified that encapsulate clinician and patient experiences with SDM: (1) Equal partnership; (2) Enhancing clinician confidence; and (3) This is different (see Figure 1 for thematic map). These themes are interrelated and comprise subthemes that address the participants’ experiences with the remote delivery of SDM in the setting of chronic stroke. Themes 1 and 3, “Equal partnership” and “This is different”, encompass the perspectives of both clinicians and patients where theme 2, “Enhancing clinician competence” incorporates only clinician content.

### 3.1 Equal partnership

This theme highlights the strength of the interpersonal relationship between the clinician and patient as the cornerstone of successful implementation of SDM. While this partnership was endorsed by both clinicians and patients, they approached it from different perspectives: clinicians from a place of looking to shift ownership to the patient and create a more equal partnership, and patients from the need to establish trust and connection as they assume more autonomy. This theme highlights the equal responsibility each participant holds in this partnership, each bringing their unique expertise, which reflects a transition away from a biomedical model where the clinician is the expert. This theme also emphasizes the importance of trust and personal connection from both clinicians and patients as a facilitator for SDM.

Clinicians expressed the need for equal partnership and patients taking on more responsibility in their recovery as goals for successful implementation of SDM. As one clinician stated, “*this whole project is really putting it more in the hands of the participants.*” However, equal partnership, under current conditions, was difficult at times. Clinicians identified necessary patient characteristics that facilitated this equal partnership. These patient characteristics include a willingness to explore therapy outside of the traditional, prescriptive approach to rehabilitation and a patient's ability to actively contribute to the conversations. As one clinician stated, “*There's also somebody that's very open to experiences and was like* ‘*OK I'm willing to follow whatever this is and then let's try and see if this is gonna work for me*’” Looking at communication, clinicians highlighted limited communication as a barrier to establishing an effective partnership:

[For one] participant, just even trying to communicate with him in writing was hard. It was hard for him to understand me and for him to write back was difficult for him. So, our meetings were usually a lot shorter than my meetings with other participants. I felt bad about that because I felt like he wasn't getting as much as the other participants did.

Lastly, clinicians highlighted the inclusion of an additional co-learner (i.e., friend or family member) to support the patient, cheer them on, and to then “bridge the gap”—essentially taking over for the clinician as the intervention ended. For clinicians, this included the expansion of ownership to include patient's friend or family member was viewed as a potential facilitator.

The patients reported increased empowerment through the SDM approach, having more control and input into their recovery. Each contributed their own expertise to this reciprocal partnership, the clinician as the rehabilitation specialist and the patient as the expert in their goals, motivations, and schedules. This was highlighted in an exchange between a patient who wanted to work on chopping vegetables and the clinician who advised him to start with a duller knife to prevent injury. The patient stated that it wasn't working and took ownership to try something different:

[After] a few tries of that, I went ahead and I started cutting real vegetables. You know…first I cut cucumbers, I tried tomatoes but they were kind of slipping, so I kind of stayed away from that… I cut the cucumber but you know beating myself up “cause I'm like they weren't consistent there were different sizes but she said ‘it's OK, but you did it.’” She said that that's the important part.

Patients highlighted the importance of trust, rapport, and connectedness within the partnership to facilitate SDM. When a patient was asked what was most helpful about the program, he stated, “*I would say… to have interaction with the OT. You know actually having someone to talk to if you have a question about something, or you're feeling something. I think that's a great thing. I really believe that that is the piece that's missing out of the medical field right now.*” Another patient stated, “*make sure you develop a good connection with your therapist. I think that's critical for the longer-term success*.” For patients, these comments underscore the need for open communication, trust, and connection for the development of this partnership and the successful implementation of SDM.

### 3.2 Enhancing clinician confidence

This theme addressed the idea that implementing a SDM approach was novel for clinicians and required training and practice beyond current experience. This includes the need for multimodal training for clinicians to gain the confidence and expertise needed to implement MI into practice. This theme emphasizes the complexity, patience, and skill training needed for successful implementation of MI for SDM. Additionally, it reinforces the third theme that SDM is a unique approach to intervention that is not adequately taught in traditional training for occupational therapists, a potential barrier for implementation of SDM within the clinic.

Some clinicians felt it was easier to revert to a more traditional, instructive biomedical model of therapy, rather than implement a more collaborative and faciliatory SDM approach. Clinicians reported barriers related to their own ability to implement MI, reporting that it was uncomfortable, demanding, hard, abstract, and required a significant amount of skill to implement. One clinician stated, “*my initial perceptions [on MI]… it demands a lot of time from the therapist. Hard, very abstract, kind of, and involves a lot of skill.*” This demonstrates MI is an unfamiliar and novel skill set to be developed and cultivated. However, confidence grew with continued practice: “*I was more savvy… with the patients as I went through*” and “*practice makes perfect, so I felt more comfortable with the last how many patients using the MI techniques because I've learned from my experiences from my first few patients.*”

Clinicians identified a need for a variety of approaches to ongoing MI training to facilitate successful implementation. These included: role playing, watching videos, getting feedback on performance, handouts, and group discussions on how to handle change talk and facilitate SDM were identified by clinicians as ways to enhance their confidence in implementing MI. This multimodal approach to MI training emphasizes the lack of confidence clinicians initially felt when applying MI in treatment. While clinicians stated that SDM doesn't “*differ from our [occupational therapy] philosophy*” in the sense that it's not that different from a standard approach in OT, the call for more intensive and varied training implies the need for education from the outset that SDM is not synonymous with more traditional OT terms such as client-centered care and therapeutic use of self.

### 3.3 This is different

This third theme encapsulates the variety of ways SDM approach differed from a traditional, biomedical model of rehabilitation. Both patients and clinicians discussed how SDM was less prescriptive in nature than a traditional approach to rehabilitation. Interestingly, SDM was different from traditional therapy for patients and clinicians in unique ways. Patients felt they had more agency and ownership over their recovery, while clinicians discussed how difficult it was to “switch” to MI-based communication.

Patients consistently expressed how “personalized” and “meaningful” the SDM approach was compared to the traditional therapy they had received in the past. This was voiced by several participants, “*it's different [than] that. You know, I have an input in what I will be doing while before you go to the outside OT and, you know, they just tell you … what you're going to do*” and “*I think the main difference is that I wasn't being led by the hand to do it. You know I was on my own I had to answer to myself for success or failure.*” Another patient stated:

In my experience with my outpatient therapy, there were some broad goals but it was OK. We sort of talked about broad goals and then the therapist … said, “OK so do this for 5 min or you know do that for 10 min” or you know, it was sort of “do this” and then I did that and then it was finished they said “all right now do this other thing.” It's a different kind of interaction.

With this different approach, though, patients expressed the concern that SDM would not have been appropriate or possible earlier in their recovery because they lacked the foundational knowledge of stroke rehabilitation they received during inpatient and outpatient therapy. This lack of perceived readiness is a potential barrier to implementation in the acute and subacute phases after stroke. As one participant stated, “*OT should be face to face when we get out of the hospital… Because it takes a while to get back into the community and back into the culture and society and stuff like that. Because you don't know, I mean, you know the world exists of course but you're kind of out of it for the first two months.*” This underscores the importance of a traditional approach early in recovery as patients may not be ready for SDM immediately after injury.

For the clinicians, the difference between the SDM approach and a traditional biomedical approach required a shift in perspective or way of thinking about therapy.

It was hard to kind of take that step back and have more of a conversation around how they could maybe incorporate [their arm] more. It was a different way of thinking. I struggled with it a little bit myself just to, you know, not throw this spiel at them.

That “struggle” was evident in the language used by clinicians, who stated that while they wanted patients to “take ownership” of their recovery rather than it being a passive process, clinicians reinforced that traditional model, evidenced by statements during the interview such as, “*[patients] do what I tell them to do*” and “*being able to carry over the program we're giving [them]*”. Expanding on the second theme's statement that new skills needed to be learned, the need for a “different way of thinking” may be a barrier to implementation for clinicians.

## 4 Discussion

Our results point to multiple areas of future work for the successful implementation of SDM in stroke rehabilitation, specifically with virtual-based UE recovery. In order to better understand each identified facilitator and barrier in the context of real-world implementation in clinical settings, we used the Consolidated Framework for Implementation Research (CFIR) (24) to further discuss each of the identified facilitator and barriers in this section. The CFIR provides a systematic approach to identify and understand various factors influencing implementation outcomes, encompassing five major domains: intervention characteristics, outer setting, inner setting, individual characteristics, and the process of implementation. Each domain contains several subdomains that offer a nuanced understanding of implementation science. We then used the Expert Recommendations for Implementing Change (CFIR-ERIC) matching toolkit (38–40). The CFIR-ERIC tool was designed to link specific implementation strategies with relevant CFIR constructs to enhance the effectiveness of implementation efforts. Table 2 outlines the barriers identified by participants per theme, aligning each with the corresponding CFIR domains and subdomains, and relevant strategies identified from the CFIR-ERIC tool.

**Table 2:** Identified themes with corresponding barriers, related CFIR domains and subdomains, and ERIC strategies for successful implementation.

Theme	Barrier	Related CFIR domains: subdomains	ERIC strategies
Equal partnership	Communication barriers	Individual domain: Personal attributes, individual states of change, knowledge and beliefs of the intervention	Conduct educational meetings Conduct local consensus discussions Identify and prepare champions
Enhancing clinician confidence	Limited training for OTs with SDM Lack of clinician confidence	Interventional domain: Adaptability, complexity Inner setting domain: Culture Individual domain: Self-efficacy, individual state of change, personal attributes	Conduct educational meetings Conduct local consensus discussions Identify and prepare champions Inform local opinion leaders Develop and distribute educational materials
This is different	Patient's lack of perceived readiness or stroke knowledge Clinician struggle to not lead, “different way of thinking”	Interventional domain: Adaptability Outer setting domain: Patient needs and resources Inner setting domain: Culture Individual domain: Self-efficacy, individual stage of change, personal attributes	Conduct educational meetings Conduct local consensus discussions Identify and prepare champions Inform local opinion leaders Conduct educational outreach visits Develop and distribute educational materials

CFIR, consolidated framework for implementation science; ERIC, expert-recommended implementation strategies.

### 4.1 CFIR-informed domain/subdomains and strategies for future implementation

First, the *intervention domain* relates to the characteristics of the intervention being implemented (24). Our results reflect several subdomains within in the intervention domain including adaptability and complexity. This study highlighted the need for this intervention to be adaptable in how it approaches training for clinicians and orientation to patients on the foundations of SDM. Clinicians with varying levels of experience and different educational backgrounds may require different modes of education and training on implementing MI for SDM. The need for SDM-specific clinician training aligns with previous research on SDM (2, 41, 42). Baker et al. (42) completed a participatory, co-design study on implementing collaborative goal setting, which included SDM, and concluded that ongoing education in the form of training modules, workbooks, and case-conferences were needed. Results from this study highlight the complexity of the UMA-R intervention with a shift away from the traditional biomedical approach to rehabilitation which may be a barrier to successful implementation. This complexity emphasizes that clinician training must consider both knowledge about the SDM theoretical frameworks and ongoing skill training to develop and maintain the skills to deliver SDM.

Strategies to address barriers at the intervention domain should then understand the unique training needs for this complex intervention and allow for an adaptive approach to initial and ongoing training based on the experience, knowledge, and needs of the clinicians. Future iterations of the intervention should develop and distribute a myriad of training materials based on educational outreach meetings prior to beginning the intervention.

From the *outer settings* perspective (i.e., the economic, political, and social or cultural context), patient needs and resources should be acknowledged. Patients who have not experienced SDM in healthcare previously may need more orientation to the expectations and power shift from more traditional biomedical models of care. Appreciating the patient's need for orientation to the SDM approach may enhance successful participation. Additionally, while not mentioned explicitly, the increased time clinicians stated they would need to feel comfortable and successful in implementing SDM led to concerns regarding time and cost barriers to implementation. Clinicians in this study expressed concerns about the feasibility of implementing MI successfully in clinical settings due to external pressures to meet goals quickly. Thus, the current structure of outpatient facilities may present barriers to implementation of SDM. Specifically, the increased time needed for MI to encourage change talk and facilitate ownership and patient decision making may be challenging to bill and document. To address these issues, it is necessary to tackle power dynamics, gain buy-in from administrators and clinicians, and resolve billing concerns. This can be addressed by engaging in local discussions, sharing knowledge, and identifying early adopters within the organization.

Looking at the *inner setting*, the need for in-depth and ongoing training for clinicians implementing MI for SDM was highlighted. Our findings suggest that current clinician knowledge and the structure of clinical settings may be a barrier for implementation of SDM. Specifically, working within the culture of clinician-as-expert is a potential barrier as patients and clinicians are equal partners in a SDM approach. Further, clinicians voiced difficulty switching from an instructive perspective to a collaborative one, reinforcing this top-down culture in rehabilitation. Therefore, quick adoption or sporadic application of SDM may not suffice within this culture; consistent practice and thorough training are crucial for successful implementation. Identifying early adopters and identifying and preparing champions for this intervention may foster change in that culture to enhance implementation.

Lastly, the facilitators and barriers related to the *characteristics of individuals* domain, were the most substantial. This includes subdomains: knowledge and beliefs about the intervention, self-efficacy, personal attributes, and interpersonal relationships. Our findings identified the importance of personal connection, openness, and trust between clinician and patient as a facilitator for SDM. This is similar to previous studies examining SDM (23, 43). The virtual format of the UMA-R intervention did not appear to be a barrier in the development of this relationship, with the exception of one patient. In general, both clinicians and patients expressed the benefits of in-person interactions, however, the development of a positive rapport was possible through virtual interactions over videoconference. There have been studies on virtual delivery of SDM where outcomes were negatively impacted by the virtual format (44). However, others have found that virtual formats did not impact to efficacy of SDM. In a study of virtual care using SDM for individuals with chronic conditions, Zickuhr et al. (45) found that a trusting patient-provider relationship, open communication, maximizing eye contact, and high quality video and audio with providers who were able to use the technology well all facilitated SDM.

Further, both clinicians and patients expressed potential barriers in regard to self-efficacy. Clinicians advocated for more intensive and varied training and need for practice to enhance their skills. Some patients expressed a lack of confidence in their own expertise of recovery with preferences more aligning with the traditional medical model where the clinicians are the experts. As one patient stated, “*I listen to the experts, professionals, and I will do [what they tell me].*” This power dynamic and lack of confidence from patients engaging in SDM is consistent with prior literature (29). Strategies to address these barriers would be conducting educational meetings and getting patient feedback to tailor education.

Looking at personal attributes, SDM may not be beneficial for all individuals at all stages of recovery after stroke. Clinicians and patients identified potential areas for consideration. First, those in the acute phase of recovery may benefit more from a traditional approach as opposed to those in a subacute or chronic phase. As one patient stated, “*Now that I'm 6 months out, I can set my own goals… but when you come out of the hospital… you need that [therapist's instructions] because you… need someone to guide you and to see you through the days*.” While SDM has been applied successfully to the individuals with acute stroke to improve UE functioning (11), it was an in-person setting rather than virtual, which may have influenced its efficacy. If the goal is to increase actual use of the affected side in daily life after stroke, there may advantages to waiting until the patient is transitioning back home. Second, patients with more severe motor impairment or significantly limited functional ability who would benefit from in-person, hands on facilitation or the use of additional technology may not be ideal candidates for virtual SDM. Last, patients with moderate to severe language processing disorders, such as aphasia, may not be able to fully participate in a SDM approach. While there are no specific strategies identified by the CFIR-ERIC tool to address the personal attributes subdomain, patient characteristics should be considered as they may be a potential barrier to successful implementation of SDM. Additionally, the identification of a co-learner or family member who could supplement the physical assistance or communication skills may be beneficial.

### 4.2 Limitations

There are several limitations to this study. The small sample size made it difficult to ascertain if data saturation was achieved, and our results cannot be generalized to the larger neurorehabilitation clinician population. Specifically, all three of the clinicians had extensive experience in stroke rehabilitation. It is possible that less experienced clinicians would provide a different perspective or have different training needs with remote delivery of SDM. All the participants for the UMA-R study expressed comfort with the use of virtual technology as the study was conducted in 2021–2022 after telehealth visits were widely used. Results may not be inclusive of underrepresented groups who experience the “digital divide” which may compound disparities already observed in SDM (46). Lastly, we did not complete member checks, which may have increased the rigor of the study and trustworthiness of the results.

### 4.3 Conclusion

This qualitative study explores facilitators and barriers for successful implementation of MI to facilitated SDM for individuals with chronic UE impairment after stroke living in the community. Key findings from this study exemplify that SDM requires equal partnership between clinicians and patients, enhanced clinician confidence, and is different from current therapy practices. Further, this study provides key components to consider for successful implementation of MI-facilitated SDM in UE stroke rehabilitation. The CFIR-ERIC tool identified several key strategies that can be integrated into future SDM implementation research including the need for educational meetings with clinicians and patients to inform them both about the unique aspects of SDM and to assess their training and educational needs, developing and distributing tailored, meaningful educational materials, as well as identifying and preparing champions and early adopters.

## Data Availability

The datasets presented in this article are not readily available because this is a qualitative study with data from recorded interviews and not available as raw data (interviews) may be identifiable. Requests to access the datasets should be directed to gjk207@nyu.edu.
